# In vitro plant regeneration and *Agrobacterium tumefaciens*–mediated transformation of *Datura stramonium* (Solanaceae)

**DOI:** 10.1002/aps3.1220

**Published:** 2019-02-07

**Authors:** Alex C. Rajewski, Kevan B. Elkins, Ashley Henry, Joyce Van Eck, Amy Litt

**Affiliations:** ^1^ Department of Botany and Plant Science University of California Riverside Batchelor Hall Riverside California 92521 USA; ^2^ Department of Botany University of Wisconsin–Madison Birge Hall Madison Wisconsin 53706 USA; ^3^ Boyce Thompson Institute Ithaca New York 14853 USA; ^4^ Plant Breeding and Genetics Section School of Integrative Plant Science Cornell University Ithaca New York 14853 USA

**Keywords:** *Datura*, green fluorescent protein (GFP), Solanaceae, tissue culture, transformation

## Abstract

**Premise of the Study:**

*Datura stramonium* is a pharmacologically and evolutionarily important plant species in the family Solanaceae. Stable transformation methodology of this species would be advantageous for future genetic studies.

**Methods:**

In vitro plant regeneration and *Agrobacterium tumefaciens*–mediated transformation techniques were developed for *D. stramonium* based on methods reported for tomato. A binary vector containing *pAtUBQ10::erGFP* was used for transformation.

**Results:**

We recovered primary transformants harboring the green fluorescent protein (GFP) transgene that resulted in expression of fluorescence in all tissues analyzed. Transformants were allowed to self‐pollinate, and two of five progeny contained the GFP transgene and displayed fluorescence identical to the primary transformants.

**Discussion:**

We have demonstrated the first stable transformation in the genus *Datura*. This is a key first step to study the genetic basis of traits in this evolutionarily interesting species.

*Datura* L. is a genus of pharmacologically important plants in the family Solanaceae. Like all members of the Solanaceae, *Datura* is notable for its production of toxic or psychoactive tropane alkaloids; however, the genus was also used extensively in the early 1900s as a model system to understand basic questions regarding hybridity, intercrossability, and species boundaries (Buchholz et al., 1935; Blakeslee and Satina, 1944; Sanders, 1948). Early studies of polyploidy were also undertaken in *Datura*, and the first production of a haploid plant was reported in *Datura stramonium* L. (Blakeslee et al., 1922). From an evolutionary perspective, the genus is also notable for its fruit type. Within Solanaceae, early‐diverging genera such as *Schizanthus* Ruiz & Pav., *Petunia* Juss., and *Nicotiana* L. possess a dry, capsular fruit. During the diversification of the subfamily Solanoideae, there was a shift in fruit type to a fleshy berry. *Datura* is located in the subfamily Solanoideae, along with fleshy‐fruited genera such as *Solanum* L. and *Capsicum* L.; however, most species of the genus *Datura* have reverted to a dry, dehiscent capsule (Knapp, 2002).

Additional detailed studies on the genetic basis of tropane alkaloid production, fruit‐type evolution, species boundaries, and other topics would benefit from the ability to stably genetically modify *Datura*. Several groups have reported and optimized various plant regeneration protocols for *Datura* spp. (Guha and Maheshwari, 1964, 1966; Sharma et al., 1993; Amiri and Kazemitabar, 2011; Amiri et al., 2011). Transient hairy‐root transformation has been reported (Payne et al., 1987; Hilton and Rhodes, 1990), and other groups have transformed *D. metel* L., but did not demonstrate stable inheritance of the transgene (Rahman et al., 2008). To our knowledge, no one has demonstrated stable inheritance of transgenes in any species in the genus *Datura*.

Here we report the adaptation of a straightforward transformation protocol developed in cultivated tomato (*Solanum lycopersicum* L.) for use with *D. stramonium* (Van Eck et al., 2006, 2018). This adaptation was successfully used to integrate a green fluorescent protein (GFP)–encoding transgene into *D. stramonium*, and the transgene was stably inherited by the progeny of these primary transformants.

## METHODS

### Germination and callus induction

*Datura stramonium* seeds were obtained in 2013 from J. L. Hudson Seedsman (La Honda, California, USA) and were grown under greenhouse conditions at the University of California, Riverside, through several generations. To aid germination, the outer seed coats of 15 seeds were removed under a stereoscope. Seeds were surface sterilized for 3 h with chlorine gas according to Lindsey et al. (2017) and transferred to medium designated 1/2 MS0 containing 2.15 g/L Murashige and Skoog (MS) salts, 100 mg/L myo‐inositol, 2 mg/L thiamine, 0.5 mg/L pyridoxine, 0.5 mg/L nicotinic acid, 10 g/L sucrose, and 8 g/L agar. Petri dishes (100 mm × 20 mm) containing 1/2 MS0 were used for germination. After 12 days, cotyledons had fully emerged and expanded, but the first true leaves had not yet appeared. The cotyledons were excised under sterile conditions, cut into ~1‐cm segments and placed adaxial side down on KCMS medium (4.3 g/L MS salts, 100 mg/L myo‐inositol, 1.3 mg/L thiamine, 0.2 mg/L 2,4‐dichlorophenoxy acetic acid, 200 mg/L KH_2_PO_4_, 0.1 mg/L kinetin, 30 g/L sucrose, 5.2 g/L Agargel [Sigma‐Aldrich, St. Louis, Missouri, USA] [pH 6.0]). The cultures were maintained at 22°C for 24 h under 100 μmol m^−2^ s^−1^ light conditions at a 16‐h photoperiod.

### Transformation and co‐cultivation

**Figure 1:**
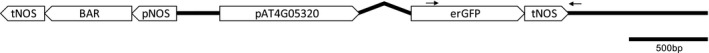
Schematic representation of the T‐DNA region of the binary vector used for transformation. This vector encodes a Basta herbicide‐selective marker (BAR) driven by the nopaline synthase promoter (pNOS) and terminated by the nopaline synthase terminator (tNOS). The endoplasmic reticulum–localized GFP transgene (erGFP) is driven by the *Arabidopsis UBIQUITIN10* putative promoter including the 5′ untranslated region (*pAT4g05320*) and is flanked upstream by the first intron of *AtUBQ10*. Transcription of the transgene is terminated by the nopaline synthase terminator (tNOS). Arrows above the schematic represent the locations of PCR primers used to amplify the GFP transgene. Left border and right border sequences of the binary vector (not shown) are located on the left and right sides of the schematic.

*Agrobacterium tumefaciens* GV3101 containing a *pAtUBQ10::erGFP* binary vector (Fig. 1) was kindly provided by Dr. Jaimie Van Norman (Van Norman et al., 2014) and grown in 25 mL of liquid Luria–Bertani medium supplemented with gentamicin and spectinomycin to an optical density measured at a wavelength of 600 nm (OD_600_) of 0.6 (approximately 48 h). The culture was pelleted by centrifugation at 4000 rpm for 10 min and resuspended in 25 mL of liquid 2% MS0 medium (4.3 g/L MS salts, 100 mg/L myo‐inositol, 0.4 mg/L thiamine, 0.5 mg/L pyridoxine, 0.5 mg/L nicotinic acid, 2 mg/L glycine, 20 g/L sucrose [pH 5.6]).

Cotyledon segments were incubated in the *Agrobacterium* suspension for approximately 5 min, then placed adaxial side down on a new plate of KCMS medium for co‐cultivation in the dark for 48 h.

### Shoot regeneration

After co‐cultivation, 70 cotyledon segments were moved to 2ZBT medium containing 4.3 g/L MS salts with Nitsch Vitamins (Caisson Labs, Smithfield, Utah, USA), 100 mg/L myo‐inositol, 20 g/L sucrose, 2 mg/L zeatin, 300 mg/L timentin, 9 mg/L phosphinothricin, 5.2 g/L Agargel (pH 6.0). Filter‐sterilized zeatin, timentin, and phosphinothricin were added after autoclaving once the medium reached ~55°C. The cotyledon segments were incubated under the same light conditions used for seed germination. Over the next two weeks, the cotyledon segments were transferred to new 2ZBT plates three times.

During this period, 23 cotyledon segments became necrotic and were discarded. The 47 surviving segments displayed callus growth and were transferred to 16‐ounce polypropylene deli containers (Fabri‐Kal, Kalamazoo, Michigan, USA) containing 1ZBT medium (identical to 2ZBT except for the addition of 1 mg/L of zeatin instead of 2 mg/L). Six weeks after co‐cultivation, the calli began to produce leaves. Over the next several weeks, the calli produced approximately 24 shoots.

### Rooting and greenhouse transfer

The survival of these plants for six weeks on antibiotic‐containing media indicated that they were antibiotic resistant and therefore transformed. To speed rooting, shoots that were 1–2‐cm tall were excised and placed on non‐selective rooting medium (4.3 g/L MS salts with Nitsch vitamins, 30 g/L sucrose, 1 mg/L indole‐3‐acetic‐acid [IAA], 8 g/L Difco Bacto agar [pH 6.0]). After one week, bacterial contamination was evident in the containers and we therefore selected nine robust shoots for direct rooting in soil in order to avoid the loss of explants to the bacterial contamination. These shoots were excised, the cut stems dipped in Rootone (Bayer CropScience, Research Triangle Park, North Carolina, USA), and placed directly in soil under a plastic dome to maintain humidity until root growth was evident.

For four weeks, the nine primary transformants did not elongate or display vigorous leaf growth as they developed roots. Three primary transformants survived this acclimatization period, while the other six were lost likely due to stressful conditions in the growth room. Approximately 4.5 months after co‐cultivation with *Agrobacterium*, the three remaining primary transformants were transferred into a greenhouse, where one further plant was lost to pest damage. The surviving primary transformants (T_0_‐1 and T_0_‐2) had vigorous growth and produced typical‐sized leaves, fruits, and seeds after their transfer to the greenhouse. These two surviving T_0_ plants were selected for further phenotyping to confirm GFP fluorescence and the presence of the transgene.

### T_1_ plants

Before the transfer to the greenhouse, the 4–5‐cm‐tall T_0_ plants began to flower despite, at this stage, having very few leaves (usually fewer than three). Flowers from two plants self‐pollinated and set fruit. The T_1_ seeds collected from fruits before the transfer to the greenhouse were small compared to wild‐type (~1 mm vs. 4 mm for wild‐type), and, upon dissection, most were determined to be empty seed coats. Five viable T_1_ seeds were produced by the primary transformants and pooled. The seed coats of these were removed, and the seeds were surface sterilized, germinated on 1/2 MS0 medium, and transferred to soil. These T_1_ plants grew normally compared to wild‐type plants, and displayed typical flowering time and seed set.

### DNA extraction and PCR conditions

Young leaf tissue (~3 cm^2^) from T_0_, T_1_, and wild‐type plants was harvested in 2‐mL collection tubes and snap frozen in liquid nitrogen. Genomic DNA was extracted according to King et al. (2014). See https://doi.org/10.17504/protocols.io.sgpebvn for a step‐by‐step protocol.

Primers to amplify 982 bp of the the GFP coding sequence were designed and checked for dimerization and deleterious secondary structure using the IDT OligoAnalyzer 3.1 (Integrated DNA Technologies, Skokie, Illinois, USA). The primer sequences were forward 5′‐CTGTCAGTGGAGAGGGTGAAGG‐3′ and reverse 5′‐TAAAGTTGCTCGAGGTACCCGG‐3′. Approximately 50 ng of genomic DNA from each plant was used to amplify a region of the GFP coding sequence using EconoTaq Plus Green 2x Master Mix (Lucigen, Middleton, Wisconsin, USA). Cycling conditions were an initial denaturation at 94°C for 2 min; followed by 25 cycles of 94°C for 20 s, 56°C for 20 s, and 72°C for 60 s; and a final extension step at 72°C for 5 min. PCR amplification of approximately 650 bp of *ACTIN* was used as a positive control. Primers for *ACTIN* were forward 5′‐GATGGATCCTCCAATCCAGACACTGTA‐3′ and reverse 5′‐GTATTGTGTTGGACTCTGGTGATGGTGT‐3′. Cycling conditions consisted of an initial denaturation at 95°C for 3 min; followed by 20 cycles of 95°C for 30 s, 55°C for 30 s, and 72°C for 30 s; and a final extension step at 72°C for 10 min. These amplicons were visualized on a 2% agarose gel stained with GelRed (Biotium, Fremont, California, USA).

### GFP visualization

Vegetative and reproductive organs of wild‐type plants, primary transformants (T_0_), and T_1_ progeny were imaged on a Leica M165 FC stereoscope (Leica Microsystems CMS GmbH, Wetzlar, Germany) using white light or, for GFP, using an 40‐nm‐bandwidth excitation filter centered at 470 nm with a 50‐nm‐bandwidth barrier filter centered at 525 nm to block chlorophyll fluorescence. All white light images were taken with an exposure time of 75–100 milliseconds, and all images for GFP fluorescence were taken with a 3‐s exposure time using a Leica D450 C digital microscope camera (Leica Microsystems).

## RESULTS

### GFP transgene amplification

**Figure 2:**
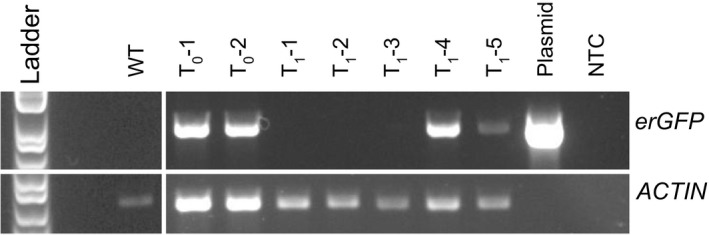
PCR amplification of a 982‐bp region of the erGFP transgene (top row) and a ~650‐bp region of the *ACTIN* control (bottom row) in a wild‐type plant (WT), two primary transformants (T_0_‐1 and T_0_‐2), five progeny of the primary transformants (T_1_‐1 through T_1_‐5), the vector used for transformation (Plasmid), and a negative control (NTC). All lanes with *Datura* DNA amplify for *ACTIN*, with the band falling between the 650‐bp and 850‐bp points on the ladder. Only the primary transformants, two progeny (T_1_‐4 and T_1_‐5), and the transformation vector amplify for the erGFP region, with a band falling between the 850‐bp and 1000‐bp points on the ladder.

Two primary transformants showed strong amplification for the expected 982‐bp PCR product (Fig. 2). Of the five T_1_ progeny assayed, three (T_1_‐1, T_1_‐2, and T_1_‐3) failed to show amplification for the GFP PCR product; however, two others (T_1_‐4 and T_1_‐5) did produce a band of the expected size (Fig. 2). Genomic DNA from one wild‐type plant, two primary transformants, and all T_1_ plants was amplified for the presence of *ACTIN* as a control for DNA quality, and all showed the expected band (Fig. 2).

### Fluorescence

**Figure 3:**
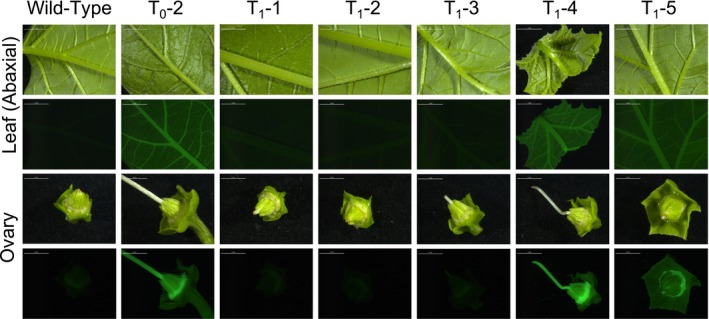
White light (first and third rows) and GFP‐fluorescent (second and fourth rows) images of abaxial leaf surfaces (first and second rows) and ovaries (third and fourth rows) from a wild‐type plant, a primary transformant (T_0_‐2), and five progeny of the primary transformants (T_1_‐1 through T_1_‐5). Fluorescence can been seen in leaf and ovary tissues of the primary transformant and two progeny plants (T_1_‐4 and T_1_‐5). All scale bars 5 mm.

The abaxial leaf surface from two of the primary transformants was imaged for GFP fluorescence and both individuals showed consistent and uniform fluorescence across the leaf epidermis; however, fluorescence was greater in the vasculature than in the epidermal tissue (Fig. 3). Adaxial leaf tissue also displayed uniform fluorescence. Tissue from all four floral whorls, immature fruits, and stem cross sections were also imaged. Stamens and pistils showed very strong fluorescence, as did nectaries and pollen. Fluorescence was very weak but detectable in the sepals and petals (data not shown). No visual evidence of mosaicism was observed. Although the endoplasmic reticulum–localized GFP transgene (erGFP) reporter construct was designed in part for its even expression in *Arabidopsis* root tissues, it is expressed in all aerial tissues of the plant. Because we grew many of our plants in soil and not on agar plates, we chose the easier, aboveground tissue for screening and did not image belowground tissue for fluorescence.

The GFP transgene was not detected in three T_1_ progeny (T_1_‐1, T_1_‐2, and T_1_‐3), and these also failed to show fluorescence above background levels. However, the two T_1_ plants that did show PCR amplification of the GFP transgene also showed fluorescence similar to the primary transformants. As observed in the primary transformants, GFP fluorescence was very strong in the stamens, pistil, pollen, and nectaries, and moderate fluorescence was consistently observed in the leaf tissue.

Wild‐type plants did not show fluorescence in leaf, stem, and most reproductive tissues. Background fluorescence was elevated in anthers and stigmatic tissue, identical to that seen in the anthers and stigmas of non‐transgenic T_1_ plants.

## DISCUSSION

Although GFP signal was clearly visible, the relatively low GFP fluorescence observed, especially in leaf tissues, could be due to a number of factors. The GFP transgene used in this study is endoplasmic reticulum–localized and driven by the *Arabidopsis UBIQUITIN 10* (*pAt4g05320*) promoter. Because of the comparatively large vacuoles in many plant cells, the endoplasmic reticulum is often pressed against the cell membrane, making the GFP signal in a single cell dense; however, across a given tissue, the signal will potentially appear more diffuse. Additionally, it has been reported that, when present in the oxidizing environment of the endoplasmic reticulum lumen, GFP folding can be disrupted and promote the formation of disulfide bonds between GFP molecules, potentially reducing fluorescent intensity (Jain et al., 2001; Aronson et al., 2011).

We have successfully regenerated transgenic plants from callus tissue of *D. stramonium* and demonstrated stable inheritance of the GFP transgene. To our knowledge, this is the first report of stable transformation and transgene inheritance of any species in the genus *Datura*, and represents an important tool for genetic studies in this evolutionarily important genus. Availability of methodology for recovery of stable transgenic lines is a critical first step for *Datura* gene function studies through approaches such as overexpression and gene editing by CRISPR/*Cas9* or other editing technology.

## AUTHOR CONTRIBUTIONS

A.C.R., A.L., and J.V.E. wrote the manuscript; A.C.R., K.B.E., and A.H. conducted the experiments; and A.L. and J.V.E. advised on experimental design.
